# “NICU Doesn’t Stop in the NICU”: Maternal Perspectives of the Impact of a NICU Experience over Time

**DOI:** 10.3390/bs16050760

**Published:** 2026-05-13

**Authors:** Celeste Poe, Leia Bonifacio, Aidan Gabriel, Natalie Jacobson, Kelli Kelley, Keira Sorrells, Richard Shaw

**Affiliations:** 1 Department of Psychiatry, Stanford University School of Medicine, Main Administration, 401 Quarry Road, Stanford, CA 94305, USA; 2 Hand to Hold, 12325 Hymeadow Dr, Ste 4-102, Austin, TX 78750, USA; 3 NICU Parent Network, P.O. Box 174, Georgetown, IN 47122, USA

**Keywords:** neonatal intensive care unit, posttraumatic stress, patient- and family-centered care, psychosocial impact, communication

## Abstract

Background: Despite the growing literature demonstrating the psychological impact of a NICU admission on parents, the longer-term adjustment to a NICU experience following an infant’s discharge is largely unknown. This study aimed to explore the NICU experience and the psychosocial trajectories of NICU graduate families after discharge. Methods: Using a qualitative design, a select group of mothers participated in qualitative interviews after completion of a quantitative survey. Interviews were completed online over a secure video platform. Participants (*n* = 21) included three groups of mothers of NICU graduates aged 2–24. Interviews ranged from 45 to 90 min, depending on the number of NICU children. The NICU care experience was explored as well as the impact of the NICU experience on parental coping, relationships, overprotective parenting, and post-traumatic growth. Results: Participants reflected on their NICU care experience and offered concrete suggestions for improvements in care. Short-term symptoms of psychological distress immediately following the NICU admission were described; however, psychological adjustment and parenting issues resulting from a NICU experience appeared to be long-term. Common themes included: (a) Effect on the Family, (b) Grief and Loss, (c) Post-Traumatic Growth, and (d) Goals for Intervention. Conclusions: These findings expand our understanding of the complexity of the NICU family experience, demonstrate the prolonged effects of a NICU admission on the family over time, and offer areas for improvement in care across the continuum.

## 1. Introduction

Approximately one out of nine newborns will experience an admission to the Neonatal Intensive Care Unit (Pang et al., 2023). Fortunately, decades of advances in neonatal medicine have led to a significant decline in neonatal death and greater rates of survival (Hug et al., 2019). With these advances, the number of infants requiring intensive care has increased, with longer lengths of stay placing infants at increased risk for adverse medical complications and poor neurodevelopmental outcomes. These outcomes include motor delays, movement disorders, impaired hearing and vision, and impaired cognitive and executive functioning (Chung et al., 2020; Song, 2023). NICU graduates have also been found to be at increased risk for mental health issues including mood and anxiety disorders as well as impairments in social and academic functioning (Chiorean et al., 2020; Woythaler, 2019). While the long-term developmental outcomes of NICU infants are well described, less is known about the parent and family perspective of the NICU experience including the management of the developmental sequelae and the psychosocial impact on parents and the family over time.

An infant’s admission to the NICU is a traumatic and stressful event that presents significant psychological challenges for the entire family (Grunberg et al., 2019). Severity of medical issues, functional impairments, financial strain, and parental role alteration all play a role in exacerbating family distress during and following a NICU admission (Grunberg et al., 2020; Lakshmanan et al., 2022; Siva et al., 2023). Experiences of shock, sadness, fear, grief, and guilt are commonly reported by NICU parents (Ireland et al., 2019; Loewenstein et al., 2019). Depression, anxiety, and post-traumatic stress are commonly reported at higher rates in NICU parents than in the general postpartum population (Bonacquisti et al., 2020; Malouf et al., 2022). NICU fathers also tend to report increased levels of distress during their infants’ NICU admissions (Cajiao-Nieto et al., 2021), with evidence of both mothers and fathers experiencing strain on the couple relationship (Reimer et al., 2023). However, despite increasing interest in psychological issues in parents, there are few studies examining outcomes beyond the first few years of the infant’s birth.

A multitude of factors associated with the NICU admission can also interfere with infant-parent bonding and parenting. Mechanisms thought to explain these findings include parent-infant interactions, parental self-competence and involvement in care, parental psychological distress, parent and infant separation, and the NICU environment (Givrad et al., 2021). Maladaptive parenting behaviors such as insensitivity and intrusiveness have also been observed in mothers of preterm infants (Hartzell et al., 2023). Siblings of preterm infants have also been found to demonstrate negative outcomes including increased anxiety and depression scores, reduced parental attention, and increased interpersonal problems (Silva et al., 2024).

Parental strategies for coping and emotional processing of the NICU experience can play a role in the overall impact of the NICU admission. Malliarou et al. (2021) for example, have found that NICU parental stress was significantly associated with engagement in substance use, spirituality and religion, and humor. In addition, Aftyka et al. (2020) found that the use of strategies aimed at seeking emotional support and positive reinterpretation were positively associated with post-traumatic growth (PTG) for fathers, while seeking emotional support, religious coping, and planning were positively associated with PTG for mothers. Parents’ perceptions of care can also affect their wellbeing. One study found that parents’ perceptions of treatment efficacy, and their satisfaction with information from medical staff was significantly associated with their levels of stress (Hames et al., 2021).

The majority of literature examining the effects of the NICU experience on families has been conducted with parents whose children are currently in the NICU or who have recently been discharged. As a result, the broad psychosocial impact on NICU graduate parents over time, including their experiences raising NICU graduates, is largely unknown. This study aimed to piece together a more comprehensive picture of the impact of the NICU family experience over time.

## 2. Materials and Methods

### 2.1. Design

A cross-sectional qualitative research design was used to explore the psychosocial impact of the NICU experience over time. A semi-structured open-ended interview explored post-traumatic stress, posttraumatic growth, overprotective parenting, and perspectives on what parents would have found helpful during and following their NICU experience. Questions were developed by experienced researchers in NICU parent mental health with feedback from mothers of NICU graduates. The interview guide included questions related to: (1) Attachment and Bonding (Can you tell me about your relationship with (child’s name)? How strongly attached do you feel to (child’s name)?), (2) Impact on Parenting (Did your child’s NICU hospitalization and/or premature birth make you more protective as a parent? If yes, can you give some examples?), (3) Post-Traumatic Growth (How have you grown from the experience of having a baby in the NICU?), and (4) Things That Would Have Helped (What additional information or support would have made you feel better prepared to take care of your child? Is there anything that might have helped you and/or your partner cope with your emotional response?).

### 2.2. Setting

Each in-depth interview was conducted over a secure video platform between a trained research assistant and participants. Participants were located throughout the country and therefore, their NICU experiences were in different hospitals. Following the interview, participants shared feedback about the questions and interview itself.

### 2.3. Participants

Participants (*n* = 21) consisted of three groups of mothers of infants who have graduated from the NICU (NICU graduates) with ages ranging from 2 to 24. Groups included young NICU graduates (ages 0–5), primary-school-aged NICU graduates (ages 6–12), and teen and young adult NICU graduates (ages 13+). Eligible participants identified as mothers aged 18 years or older, who had given birth to infants previously admitted and discharged from the NICU. Participants were recruited from non-profit support organizations for NICU parents, with collaboration from NICU non-profit organizations Hand to Hold and NICU Parent Network. Sample size was determined with consideration for recommendations based on the study’s aim, sample characteristics, and the quality of the data (Bekele & Ago, 2022). Table 1 provides information on participant demographics.

### 2.4. Procedure

Approval for the present study was obtained from the Stanford University Institutional Review Board. Participants were contacted by the study coordinator via phone or email, were briefed, provided informed consent, and asked to complete a 15–20 min questionnaire. The present study represents findings from the 45 min to 90 min interview that followed for select participants. Interviews were recorded and transcribed anonymously into Stanford Medicine Box (HIPAA compliant) and erased post-transcription.

### 2.5. Analysis

Data analysis involved a hybrid reflexive thematic analysis approach in which both a top-down theoretical approach and a data-driven approach were used. Detailed accounts of the NICU experience on parents and the family over time were deeply examined (Swain, 2018). Double coding by two researchers familiar with NICU family experiences was used to confirm inter-coder reliability. Transcribed data was first reviewed, and a coding index was created. A rigorous and iterative process, the thematic analysis approach began with four rounds of data review, with initial codes generated and relevant data collated for each code. Both coders then independently organized codes into potential themes and gathered relevant data within each potential theme. Across various discussions, coders agreed on the majority of themes while themes related to overprotective parenting and grief and loss were discussed the most. Coding decisions were compared, and discrepancies were discussed in a reflexive process to reach agreement between the coders. Themes were named and defined, and a thematic map was created as ongoing analysis was completed in order to refine the conceptualization and articulation of each theme and the overall narrative of the data (Braun et al., 2023).

## 3. Results

Mothers of NICU graduates discussed high levels of psychological distress during and immediately following the NICU admission. Despite appearing to decrease over time, symptoms did not disappear completely, demonstrating long term effects on the family including parenting and feelings of loss. Mothers also reported post-traumatic growth and shared important perspectives on their care experience. Four major themes emerged in the present sample: (a) Effect on the Family, (b) Grief and Loss, (c) Post-Traumatic Growth, and (d) Goals for Intervention. While experiences were similar across groups, anxious and/or overprotective parenting appeared to most commonly occur in the first few years following discharge and to decrease in intensity and frequency over time.

Effect on the Family

Mothers of NICU graduates across age groups described the various ways in which their NICU experience affected all members of the family, altered family relationships, and influenced parenting behaviors.

Effects on Parents, the Couple, and Siblings

The first subtheme highlighted the effects of the NICU experience on various members of the family, with many mothers describing symptoms of traumatic stress. One mother of a 6-year-old NICU graduate reflected on her time in the NICU.

It wasn’t until later that I feel like I recognized that some of this is just PTSD and I don’t think I recognized those signs until after the fact … I remember going to see [infant], and you got off the elevator, and before you got to the scrub room there was a bathroom and I remember being sick in that bathroom so much just because I was so nervous.

Many parents of NICU graduates reflected on the ways in which the NICU experience affected their other children. One mother of an 8-year-old NICU graduate illustrated how siblings can be affected by the complex medical needs of NICU graduates after discharge.

When [infant] had her trach … it was a two-man job. During this whole process it was like ‘okay [older sibling], go over there, go over there, go over there.’ There was one time that we were changing out the trach and she came and jumped on my back, and I yelled at her and was like do not do that, you have to wait … and she started crying and said, ‘I wish I had a trach,’ which I mean, of course, broke my heart.

Several mothers also spoke about the effect that a NICU admission can have on the couple relationship which often included differences in coping during and following the NICU stay. One mother of an 18-year-old NICU graduate described the differences in ways she and her husband at the time approached their infant’s NICU admission.

I was married. We’re no longer married—maybe partly because of this … He just was not supportive of me, and I became very angry about that. Like, he never went to the NICU to see her and she was there for three weeks … I stayed in the hospital for five days; he would come to visit me every day and every time I would say, ‘Did you go by and see the baby?’ … and he said, ‘No, I will on my way out’ … and he never did. That, to me, it was like … it was the beginning of the end, I think.

Impaired Bonding

Many mothers described the negative impact of a NICU admission on the child–parent relationship including their identity as parents and ability to effectively engage in bonding and promote attachment. A key component of building early attachment and bonding with an infant is the reciprocal nature of the relationship, or the back and forth of cues and responses between an infant and caregiver (Wright & Kong, 2023). One mother of a 2-year-old NICU graduate described her delayed ability to bond and connect with her child given developmental limitations.

 … when he came home, he was 37 weeks gestational age, adjusted, so it felt like a really, really long time (and this was harder for my husband than for me I think) before he provided any positive reinforcement or encouragement. He didn’t smile before he was four months old. All he did was scream at the beginning because he was in an age deficit essentially. As our first child, that was pretty difficult as it felt like a very long period of darkness with no positive reinforcement.

Overprotective Parenting

Nearly all mothers discussed the ways in which their infant’s NICU admission affected their parenting, including descriptions of anxious, intrusive, or overprotective behaviors due to excessive health or developmental concerns. One mother of a 17-year-old NICU graduate described the level of ongoing worry that affected her parenting.

I wanted to bubble wrap him. I wouldn’t let him climb trees unless I was holding him. I didn’t want him walking on concrete because I was afraid he was going to fall and hurt himself and get a scratch so I was more overprotective than I would have been otherwise had he been born full-term.

Another mother of a 5-year-old NICU graduate described difficulty with letting her child go places without her.

I am definitely overprotective. If she’s here, I’m okay … I’d love for her to have the free lifestyle I had growing up, but letting her go into someone else’s care, I almost feel like there’s this weird dynamic where I never felt like she was my kid at the NICU.

Grief and Loss

The second major theme involved mothers’ descriptions of grief and loss as a result of the NICU admission. Mothers of NICU graduates across age groups shared these experiences.

Loss of Identity

Nearly all mothers described experiences of losing children, loss of plans for the future, changes in relationships with family and friends, changes in jobs or career trajectories, and alterations in spirituality or religion. One mother of two 18-year-old twin NICU graduates described the change in her career trajectory.

I had to resign from my job. Part of the resentfulness that comes from having them is I had to give up my career, and … that’s been a hard, hard one. I just went back to work six years ago, and I’m still not at a point where I want to be and I’m kind of frustrated by that.

A few mothers described doubt, anger, and a sense of disconnect from their spirituality or religious beliefs. One mother of an 8-year-old NICU graduate discussed her spiritual struggles.

I’ve felt mad before. Like religion is a part of my life, and especially when she turned blue when she was two, I remember just sitting and sobbing to my husband and saying, ‘I am so mad at God right now’.

Loss of the Imagined Experience

Nearly all mothers spoke about the ambiguous loss of the imagined pregnancy, birth, or postpartum experience. For example, one mother of two NICU graduates, ages 17 and 19, expressed:

The most challenging parts were probably the emotions that went along with not having the experience that I thought it would be from the standpoint of pregnancy, having a baby, all the dreams and visions I think a parent would expect and dream of.

Missing out on meaningful moments or anticipated milestones such as holding one’s infant for the first time following birth or being able to breastfeed were also discussed. One mother of a 5-year-old NICU graduate explained:

Everything I had worked so hard for and towards, everything was stolen. I had all these things in place, and all of it got ripped away from me. I had a doula, an acupuncturist, a photographer … and then when I had her, no one knew … I think the things I was robbed of will be the things that will always get to me … and the way things went down, I didn’t connect with her at all for weeks. I couldn’t hold her for weeks. In our minds … you have the beautiful birth story that you’ve always wanted. All of these beautiful moments. And none of that happened … 

For many NICU families, plans to have additional children can be cut short due to the trauma of the NICU experience or potential safety concerns for the mother or fetus. For example, one mother of a 7-year-old NICU graduate shared her family’s decision to stop having children.

… Our physician said it was a good idea not to try because [first baby] was small and [second baby] was even smaller and the next one would probably be even smaller so yes it did change our mind at having other children.

For families who do choose to have more children after a NICU experience, anxiety can impair feelings of joy or excitement when having a baby. One mother of a 6-year-old NICU graduate described her concerns during her subsequent pregnancy.

It just made me more nervous when I was pregnant with her. Like every ultrasound, I didn’t feel happy. I felt a little scared. Like, is this gonna be bad? And so, it was hard to like, enjoy it.

Post-Traumatic Growth

In addition to adverse consequences following experiences of traumatic events, it is common for individuals to report positive outcomes or experiences as the result of their traumatic experience (Koivula et al., 2022). Mothers of NICU graduates across age groups discussed personal areas of posttraumatic growth.

*Intrapersonal Growth*

Most mothers reflected on areas of intrapersonal growth including feelings of pride, strength, resilience, newfound purpose, and a more positive outlook on life. One mother of two NICU graduates, 22 and 24 years old, stated:

I guess it confirms there’s a lot more we can draw on when we need to. A lot of strength.

Similarly, a mother of a 6-year-old NICU graduate expressed:

I definitely feel like there’s not a lot that can stop me … things don’t faze me as much. … 

*Interpersonal Growth*

Mothers also described growth in their interpersonal relationships and relational capacities including increased empathy, compassion, and an increased ability to accept help from others following their NICU experience. One mother of a 2-year-old NICU graduate noted that she was proud of the ways in which she and her husband were able to work together while their infant was in the NICU.

That we leaned on each other. I felt like [father of baby] and I were really able to talk to each other versus pushing each other away.

*Spiritual Growth*

Some mothers also described ways in which they felt their spiritual connection or relationship with God was strengthened as a result of their NICU experience. For example, the mother of a 6-year-old NICU graduate expressed:

There were moments in the NICU where we realized how critical things were and how precious life is and how quickly it can be gone … Not as much in the moment, but when I look back and see how things played out and the timing of things, I definitely had a sense that you know, we were being watched over and that there were other hands at work more so than any other experience in my life.

*Paying it Forward*

Several mothers reported a desire to find opportunities to give back, pay forward, or provide the support they needed during their infant’s NICU admission. For many of the mothers, this involved career changes or engagement in volunteer peer mentorship. One mother of two 15-year-old NICU graduates explained how her career changed following her family’s NICU experience.

Nothing just happens by magic and I know we do have a purpose greater than I can comprehend. I mean, when we first started the non-profit, I was just like I want to help parents at home to get the needed medical equipment and now we have this national network of 40+ organizations, we’re speaking at conferences, I mean I never thought I’d have my name written as a co-author in a medical journal. That’s just … there are things out there that you can’t even dream of.

Goals for Intervention

Mothers of NICU graduates across age groups discussed their care experiences before, during, and after their NICU stay and outlined areas for improvement.

*Better Preparation*

Many mothers highlighted the desire for better preparation of NICU families for what they should expect during and after their NICU stay. For example, one mother of an 18-year-old NICU graduate explained:

It would have been nice to have had someone explain the experience of the NICU. I didn’t know anything about it. No one told me oh you’re gonna have to scrub—no one told me what it was going to look like. I had no idea.

Many mothers specifically noted the lack of proper preparation for the transition home from the NICU. One mother of a 6-year-old NICU graduate described how the unrealistic expectations made her question herself as a parent.

I think what would have been helpful is someone who would have been able to tell us what to expect. We just had a lot of people insisting like ‘you’re taking home a healthy newborn, pretend he’s a newborn, he’s on a schedule.’ That was not true. He didn’t sleep for two years, he puked all the time because he had to have a feeding tube down his throat for so long, and for a year and a half I thought it was just because we weren’t doing a good job as parents.

*Clear and Compassionate Care*

The need for more compassionate care and clear communication from staff was also highlighted. Mothers noted the importance of feeling seen and understood, respected, and communicated with in ways they found helpful. One mother of a 4-year-old NICU graduate described her observations of judgmental and differential care provision.

The nurses … if you didn’t go see your baby, I would hear them talk about the moms. Some of the moms would definitely look like they were experiencing postpartum depression so they literally couldn’t come in, but the nurses wouldn’t see it like that. If you were there all the time the nurses would treat you better than if you weren’t there.

Mothers also discussed the importance of providers being thoughtful and intentional in their care. For example, one mother of a 6-year-old NICU graduate described how peer support was not provided at the most appropriate time given her infant and family’s circumstances.

The hospital I delivered at sent a mom in who had a 6-year-old who was born early, like the day before I even got to go see [my child], and they are trying to give me this pep talk, like here’s the light at the end of the tunnel, this can be you. But in that time period, I just didn’t feel like I was ready to hear that. It was super well intentioned, and now I see where they were coming from, but in that emotionally flooded point I wasn’t able to receive that. I didn’t even know if he was going to live through the week and I hadn’t even held him yet … it was just a lot of steps ahead of where I was at.

Another mother of a 5-year-old NICU graduate expressed the importance in clear and realistic communication from healthcare providers.

Sometimes the brutal truth is what you need … I feel like I would’ve rather a doctor sit me down and say … the conversation that I’m about to have with you is going to be very difficult to hear … you may not retain all this information, but I need to let you know that you, you will be in the hospital for another 12 weeks, you’re here because you know you’re unstable, baby’s unstable because you’re unstable and you could have the baby at any time. You may not have the baby, well, you wanted, we’re not going to be able to follow your birth plan.

*Resources*

All mothers discussed the resources necessary to properly support NICU families including mental health support, lactation support, family advocates, support specifically for fathers, and opportunities to connect with other parents through groups or peer mentorship. For example, one mother of a 5-year-old NICU graduate expressed:

You know, I wish I knew about NICU groups. I wish I knew about some of the NICU forums that there were, that there was an outlet to talk to other people … I would’ve literally given a limb if there was someone that I could have talked to that had experienced anything, like, any of it.

In addition to many mothers noting the importance of in-person services, mothers also discussed the value in offering services as early as possible. For example, one mother of two 15-year-old NICU graduates explained:

I didn’t get the maternal [mental] health specialist until 6 months after [one of the triplets] died so they were almost two years old, so once I got that, coupled with being on the right medication for me, that’s when the trajectory started to change for me, but I didn’t know that existed, that there were maternal [mental] health specialists, but that was my OB referring that to me almost two years after but I feel like she should have done that as soon as I knew I was going to have a high risk pregnancy.

Additionally, one mother of a 2-year-old NICU graduate emphasized the significant gap in support for fathers.

I mean, that is really traumatic, and nobody really asked him, ‘hey dad, how are you feeling about it?’ I got so many questions like, ‘hey mom how is your scar feeling? Are you producing milk? Are you doing this and that?’ but nobody says anything to the dads, and I feel like that’s really hard.

Additional quotes and interpretations can be found in Table 2.

## 4. Discussion

### 4.1. Summary and Interpretation

The current study is one of a few to portray such a complex picture of the NICU family experience over time. The wide age range among experiences and findings related to overprotective parenting and post-traumatic growth expand our knowledge of the NICU parent trajectory. Several factors appeared to impact the NICU experience during admission, and while appearing to improve over time, overall family psychological adjustment following a NICU admission has the potential to affect family relationships, parenting, and feelings of grief and loss. Direct maternal perspectives on the NICU experience also shed light on ways to improve intervention and make resources available to NICU parents across the care continuum.

The evidenced intensity of parental psychopathology in the first few years following a NICU admission is consistent with previous research (Haemmerli et al., 2020; Rodrigues et al., 2024; Schmöker et al., 2020). However, the current study highlighted that feelings of grief, loss, and guilt can be more enduring, in some cases lasting as long as two decades after the NICU hospitalization. In addition to feelings of traumatic stress and grief, studies examining the impact of the NICU experience on parents also describe post-traumatic growth in a number of areas (Gong et al., 2023). We had similar findings in our sample with descriptions of perceived areas of growth including intrapersonal and interpersonal strengths and a desire to pay support forward. It should be noted that given the study sample, greater socioeconomic resources are likely to increase the ability to be involved in volunteer and entrepreneurial opportunities. Literature on parental distress and coping in the NICU has examined the complex role of religion and spirituality (Brelsford & Doheny, 2022). Findings from the present study suggest that some parents may have moments of doubt and feel frustration or anger with God while others may turn to God or their spirituality for comfort during moments of high distress.

The impact of the NICU experience on family relationships appeared to occur in multiple ways. For example, consistent with previous studies (Baraldi et al., 2020), while some mothers reported strengthened couple relationships following a NICU admission, quite a few mothers described the strain the NICU admission placed on the relationship with their partner. The NICU admission also appeared to affect the child–parent relationship. Many mothers indicated early impaired bonding and relating with their infant as a result of the NICU experience, and while the majority reported a greater appreciation for their child, many discussed a tendency to engage in overprotective, anxious, and intrusive parenting. These maladaptive parenting behaviors resulting from a NICU admission have been previously described (Shaw et al., 2023; Wang et al., 2021). Additionally, findings demonstrated various experiences of ambiguous loss (Boss, 2010), as mothers described the loss of the imagined baby, perinatal or parenting experience, and career trajectories.

Consistent with previous literature on supportive interventions for parents in the NICU, mothers of NICU graduates described the need for interventions to be available early, in-person, and at important transitions across the care continuum (Erdei et al., 2021; Furtak et al., 2021). Essential supportive services include lactation support, mental health support, advocacy, support for fathers, and opportunities for parents to connect with other NICU parents through support groups and peer mentorship (Adama et al., 2022; Galea et al., 2022). Finally, mothers shared the importance of open, compassionate, and thoughtful care which is consistent with findings emphasizing the importance of communication in care settings (Labrie et al., 2021).

### 4.2. Limitations

Although the qualitative data from the current study offers direct in-depth descriptions of maternal perspectives of the impact of the NICU experience on the family, a more comprehensive analysis will be achieved once integrated with quantitative data also gathered. The study, which only offered a maternal perspective, included a sample of convenience, with mothers recruited from peer support organizations who have likely had opportunities to reflect and process their NICU admission. The perspective of fathers, who have unique roles and expectations in the perinatal period and in the NICU, would have also strengthened this study. Additionally, the wide range in age of NICU graduates should be considered, as it is likely that this limited mothers’ ability to accurately recall their NICU experience or may have altered their perspective on this time in their life. This wide range in age may also affect the reliability and comparability of experiences given the likelihood of medical, structural, and cultural changes in the NICU setting over time. Homogeneity of participants was also a limitation. While participants and their NICU graduate children varied in age, the majority of them were White, highly educated, and English-speaking, offering a valid but narrow perspective and experience of more resourced families. Findings would be broadened and contextualized by a more heterogenous sample.

### 4.3. Implications

Findings from this study expand our understanding of the NICU family experience, highlighting the care experience over time, demonstrating the psychosocial impact on the family unit, and exemplifying the critical need to adhere to principles of family-centered care. Throughout the NICU care experience, from admission to post-discharge, families are constantly adjusting to new expectations, transitions in their parental role, and managing feelings of distress and loss. Beyond infant outcomes, parental psychological adjustment to a NICU experience can be shaped by the parental role in the NICU setting and the infant-parent relationship, parents’ relationships with NICU staff, and parents’ perceptions of care.

The psychological impact of a NICU experience including parental trauma, grief, hope, and impaired parental functioning can be complex and incessant, potentially impacting typical child development and subsequent reproductive experiences. Follow up and screening for psychosocial needs in NICU families should be considered in the NICU and well after NICU discharge including the child’s social and emotional development, parenting support, and couple and family relational dynamics. NICUs and NICU follow-up settings should invest in embedded NICU psychologists who specialize in meeting these complex psychological, emotional, and relational needs. NICU families should also be offered opportunities to connect with other NICU parents through support groups or NICU parent mentors.

The NICU experience begins early and can last well beyond discharge. Families with fetal diagnoses, high-risk pregnancies, babies in the NICU, and in outpatient follow-up and primary care settings should receive clear and accurate information and access to resources for whole-family wellness including fathers. During what can be a very challenging time for all members of the family, care that is trauma-informed and rooted in empathy and compassion can support parents in feeling safe and supported. Training should be provided to all NICU staff on the basics of NICU parent mental health, NICU infant bonding and attachment, and trauma-informed care and communication. Implementation of family-centered care practices that emphasize the value of the parental role is essential in building a strong foundation for adaptive parental functioning and infant-parent bonding and attachment. NICUs would benefit from education and partnership with the NICU Family Centered Care Taskforce (Balasundaram et al., 2025) for resources and support around improving these practices with their patients and families.

Families may be navigating the NICU journey and parenthood for the first time and would benefit from support in anticipating significant transitions across the continuum of NICU care. Interdisciplinary communication across settings is essential for collaboration in meeting these needs including provision of timely and transparent information. Care team members such as social workers, NICU psychologists, or case managers may be assigned to provide support to parents during times of transition. Use of transparent and compassionate communication and guidance can support parents in having realistic expectations, managing anxiety, holding hope, and engaging in adaptive parenting practices throughout their child’s development. Parents should be considered partners in care with encouragement to participate in rounds and care conferences, offer opinions, and share concerns. Multidisciplinary discussion and collaboration concerning psychosocial needs is critical in providing true family-centered care.

## 5. Conclusions

NICU graduate families are a vulnerable population that often go overlooked following a discharge from the NICU. However, the NICU experience is greater than the time from admission to discharge and has the potential to lead to impaired parental psychological and relational functioning, maladaptive parenting practices, and unresolved loss. This study illustrates the psychosocial impact of a NICU experience on the family over time, offering insight into targets for intervention, areas for improving care, and strategies for optimizing NICU family developmental, social-emotional, and relational outcomes throughout the life course.

## Figures and Tables

**Table 1:** Demographic Information (*n* = 18; 3 participants with missing demographic data).

Mean age (years)	40.6
Highest Level of Education	
Less than High School	0%
High School Graduate	0%
Associate Degree	6%
Bachelor’s Degree	33%
Graduate/Professional Degree	61%
Current Relationship Status	
Single	0%
Committed Relationship	83%
Other	17%
Household Income	
<$20,000	0%
$20,000–$39,999	0%
$40,000–$59,999	6%
$60,000–$79,999	11%
$80,000–$99,999	11%
>$100,000	67%
Racial/Ethnic Background	
African American/Black	6%
Asian/Asian American	0%
South Asian	0%
Mexican/Mexican American/Chicano	11%
Native American	0%
Other Hispanic/Latino	0%
White	83%
Religious Background	
Christian	39%
Catholic	33%
Jewish	6%
Hindu	0%
Muslim	0%
Buddhist	0%
Other	22%
Number of Pregnancies (mean)	2.5
Fertility Treatment	40%
Presence of Parent Mental Health Issues	67%
Emotional Support Services Used by Parent	
Peer Mentor	39%
Parent Support Group	22%
Individual Psychotherapy	72%
Group Therapy	6%
Marital Therapy	11%
Family Therapy	6%
Psychotropic Medications	33%
Infant’s Gestational Age at Birth (weeks; mean)	27.9
Infant Birthweight pounds	3.25
Infant Gender at Birth	
Male	56%
Female	44%
Age of NICU Graduate	
1–7 years	47%
8–14 years	23%
>14 years	29%
Number of Siblings in Family	
None	22%
1	56%
2	22%
Number of Hospitalizations of NICU Graduate	7.5 (range 1–7 times)
Presence of Learning Issues	22%

**Table 2:** Themes, Subthemes, and Selected Quotes.

Theme: Subtheme	Quote	Interpretation
Effect on the Family: Effects on Parents, the Couple, and Siblings	“…when we go to the hospital, I’ll have an instant breakdown just in the parking lot before we even go in…I am currently pregnant, and I had to get an ultrasound recently, and even just being in the building gave me a full-blown panic attack. I had to sit down, and I was terrified.”	Mother of 2-year-old NICU graduate describing how triggering subsequent visitations to the hospital can be following a NICU experience.
Effect on the Family: Impaired Bonding	“We had in-home feeding therapy, at-home physical therapy, play therapy, but then we also had to go to ophthalmology, pediatrician, in-home pediatrician, the GI doc, the cardiologist, pulmonologist, it was constant. It was like we were home, but I was the one running the NICU now. In some ways I think I resent the fact, I mean I’ve gotten over it now, that I didn’t get to be the parent, I just had to be this caregiver and caretaker and it never stopped until they were around 2.”	Mother of 18-year-old NICU graduate twins noting the difficulty with being able to take on the role of parent after discharging from the hospital.
Effect on the Family: Overprotective Parenting	“…and to this day she goes to the potty at night, I rub her belly with Vaseline. I look at her from head to toe, in between toes, in her ears, up her nose, down her mouth, I mean it’s crazy – like I’m that mom.”	Mother of 4.5-year-old NICU graduate detailing the increased level of attention and care paid to her daughter as a result of her NICU experience.
Grief and Loss: Loss of Identity	“Initially, I reached out to people. But then after the first week or two people stop checking in and so then it becomes very lonely and isolating.”	Mother of 3.5-year-old NICU graduate describing the loss of social relationships during and following a NICU experience.
Grief and Loss: Loss of the Imagined Experience	“You know, my dad was saying he doesn’t understand why it’s so upsetting because she’s healthy, she’s beautiful, but it’s like a scar—it’s a scar on your heart. You’re grieving something that you don’t really understand. I mean there are people whose babies don’t make it, so you’re grieving the loss—and in some ways at least it makes sense as to why you’re grieving. But like, why am I grieving? My kid is 18. She’s healthy. I’m proud of her. But I think you’re grieving what you thought your experience was supposed to be.”	Mother of 18-year-old NICU graduate describing experiences of ambiguous loss even when an infant survives the NICU.
Post-Traumatic Growth: Intrapersonal Growth	“Definitely stronger than I thought, a bigger advocate than I ever thought…I think the advocate piece–being able to stand up for yourself…”	Mother of 7 -year-old NICU graduate reflecting on the ways in which her strength and ability to advocate for herself during the NICU admission surprised her.
Post-Traumatic Growth: Interpersonal Growth	“I think I’m a better person. A better listener. More empathy. You know, you can put yourself in other people’s shoes. Learning to be more supportive. Or at least, allowing people to share. I think that has made me a better person.”	Mother of 18-year-old NICU graduate expressing increased capacity to demonstrate empathy and support for others following her family’s NICU experience.
Post-Traumatic Growth: Spiritual Growth	“We had a strong faith and relied on God tremendously…I’ve become stronger in my faith, relying on God more. He carried us through a lot of it. I think he’s more in control than we thought.”	Mother of 7-year-old NICU graduate expressing growth in her trust and ability to rely on God following the NICU admission.
Post-Traumatic Growth: Paying it Forward	“I’m a postpartum peer coach. So, moms from all over the region can call because, you know, either they’ve had a premature baby or just stressed about parenting or, you know, usually a special needs kids. Then I get paired with them and then I talk to them once or twice a week and just hold space for them…so that’s something I’m really proud of.”	Mother of 4.5-year-old NICU graduate describing the fulfillment she gets from acting as a peer coach to other NICU parents.
Goals for Intervention: Better Preparation	“You know, I mean, they teach you, they make you watch videos about SIDS…they’re not making you watch a video about how to transition successfully or, you know, those kinds of things”.	Mother of 4.5-year-old NICU graduate admitting that while there is some preparation prior to discharge, most of this preparation is focused on general safety concerns for newborns.
Goals for Intervention: Clear and Compassionate Care	“I think it would have helped a lot if we could have seen pictures of other kids thriving with g tubes and trachs because when you hear the word trach you think of like “oh God, you can’t have a normal life like that”…instead of just getting all of the really terrible messages… it would have been really helpful for someone also to say “hey, here’s how you can take her to the playground” and just bring awareness to the fact that that’s a choice. So normalization really.”	Mother of 7.5-year-old NICU graduate explaining how helpful it would have been for providers to have found a balance between cautionary guidance and supportive normalization.
Goals for Intervention: Resources	“We were really lucky. At [hospital] there was a therapist down the hall that was free. It was incredible…and I think the best part was I didn’t have to leave my child to do that. I could literally walk 50 feet down the hall and be there. And it was free, so I didn’t have to stress about the calculations of you know the cost…”	Mother of 6-year-old NICU graduate expressing the importance of accessible in-person mental health support.

## Data Availability

Data will be made available upon reasonable request and after ethical approval.
